# Association of red blood cell distribution width and outcomes in patients with mantle cell lymphoma

**DOI:** 10.1002/cam4.2155

**Published:** 2019-04-13

**Authors:** Yi Miao, Xiao-Hui Zhou, Jing-Jing Guo, Qian Sun, Ke Shi, Jia‐Zhu Wu, Hua‐Yuan Zhu, Li Wang, Lei Fan, Wei Xu, Jian‐Yong Li

**Affiliations:** ^1^ Department of Hematology The First Affiliated Hospital of Nanjing Medical University, Jiangsu Province Hospital Nanjing China; ^2^ Key Laboratory of Hematology of Nanjing Medical University Nanjing China; ^3^ Collaborative Innovation Center for Cancer Personalized Medicine Nanjing China

**Keywords:** Mantle Cell International Prognostic Index, mantle cell lymphoma, prognosis, red blood cell distribution width, survival

## Abstract

Red blood cell distribution width (RDW), which measures the range of variation of red blood cell volume, has been explored as a prognostic factor in multiple types of cancer. However, the role of RDW in mantle cell lymphoma (MCL), a rare type of non‐Hodgkin lymphoma with poor outcomes, remains to be determined. Therefore, we investigated the prognostic role of RDW in MCL. We found that 21 of 76 MCL patients (27.6%) had an abnormally elevated RDW (>15.7%). Abnormally elevated RDW was significantly associated with presence of B symptoms (*P* = 0.0020), elevated lactate dehydrogenase (LDH) (*P* = 0.0010), higher leukocyte count (*P* = 0.0345), higher simplified Mantle Cell International Prognostic Index (sMIPI) (*P* = 0.0194), and lower level of hemoglobin (Hb) (*P* < 0.0001). It was marginally associated with increased C‐reactive protein (*P* = 0.0862). RDW was significantly correlated with Hb level (*r* ^2^ = 0.42) and LDH level (*r* ^2^ = 0.19). 15.8% was determined as the best cutoff of RDW in predicting the survival outcome by the X‐tile software. Survival analysis revealed that high RDW (>15.8%) predicted shorter progression‐free survival (PFS) (hazards ratio [HR]: 3.14; *P* = 0.0005) and shorter overall survival (OS) (HR: 4.04; *P* < 0.0001). High RDW independently predicted both shorter PFS (*P* = 0.0493) and OS (*P* = 0.0118). RDW also improved the prognostic stratification based on sMIPI. In conclusion, our study identified RDW as a novel prognostic factor of clinical feasibility in the prognostication of MCL.

## 1 INTRODUCTION

Mantle cell lymphoma (MCL) is a rare type of non‐Hodgkin lymphoma with poor outcomes.^1^ The standard therapeutic approach for MCL remains undefined. In clinical practice, young fit patients are usually treated with aggressive induction therapy containing rituximab and cytarabine followed by consolidation stem cell transplantation and rituximab maintenance, while older patients are treated with chemoimmunotherapy followed by rituximab maintenance.^1^ Although the outcomes of patients with MCL have improved with currently available treatments, the prognosis of patients with MCL is heterogeneous. Identification of factors associated with clinical outcomes could facilitate risk stratification and improve individualized treatment. The prognostic index Mantle Cell International Prognostic Index (MIPI), which is based on age, performance status, lactate dehydrogenase (LDH), and leukocyte count, can classify patients with MCL into three groups with different clinical outcomes.^2^ Simplified Mantle Cell International Prognostic Index (sMIPI) also has similar discriminative power in MCL patients.^2^

Red blood cell distribution width (RDW) is a marker that measures the range of variation of red blood cell volume. And in clinical practice, RDW values are usually used to determine the cause of anemia.^3^ High RDW values can be observed in cases of iron‐deficiency anemia or folate‐ and vitamin B12‐deficiency anemia, but not in thalassemia. Additionally, elevated RDW has been reported to be associated with other pathophysiological states including cardiovascular events, cancer, and autoimmune diseases.^4^ Moreover, RDW has been identified as a prognostic factor in several types of cancer, including some hematological malignancies.^5^ However, the association of RDW with survival outcomes in patients with MCL is unknown. In this study, we assessed the association of pretreatment RDW with progression‐free survival (PFS) and overall survival (OS) in a cohort of patients with MCL.

## 2 MATERIALS AND METHODS

### 2.1 Patients and baseline variables

Seventy‐six consecutive newly diagnosed MCL patients between January 2009 and July 2017 were included in our study. This study was approved by the ethnical committee of The First Affiliated Hospital of Nanjing Medical University. For cases included, the presence of IGH‐CCND1 translocation and/or Cyclin D1 expression by immunohistochemistry was mandatory for the diagnosis of MCL.^6^ Clinical variables and laboratory data were obtained from medical records. The baseline variables collected included age, gender, Ann Arbor stage, B symptom, Eastern Cooperative Oncology Group performance status, extranodal sites, LDH, hemoglobin (Hb), total bilirubin (TBIL), ferritin, and C‐reactive protein (CRP). Anemia was defined as a Hb value less than or equal to 120 g/L for men and women over 50 years of age, and less than or equal to 110 g/L for women under 50 years of age. sMIPI was calculated for all the patients.

### 2.2 RDW measurement

RDW value at the time of diagnosis was collected. RDW was automatically analyzed by the Sysmex XE‐2100 hematology automated analyser. The normal reference for RDW in our hospital is 10.0%‐15.7%.

### 2.3 Statistical methods

The Student's *t* test or Mann‐Whitney *U* test was used to compare continuous variables between two groups. D'Agostino‐Pearson omnibus normality test was carried out to examine if the values came from Gaussian distribution. If the values passed normality test and equal variance test, we used Student's *t* test, otherwise Mann‐Whitney *U* test was used. Categorical variables were compared using Fisher's exact test. Spearman correlation analysis was applied to evaluate the correlation between RDW and other continuous variables. For CRP, four cases had a CRP value under the lower detection value, thus these four cases without an exact CRP level were not included in correlation analysis. Survival was defined as time from diagnosis to death or last follow‐up. PFS was defined as time from diagnosis to progression, death or last follow‐up. Survival curves were constructed using Kaplan‐Meier method, and log‐rank test was used to compare the difference. Multivariate analysis was done by multivariate Cox model. The cutoff of RDW for survival analysis was determined by using the X‐tile software.^7^ Statistical analyses were performed using Graphpad Prism 6 (GraphPad Software, San Diego, CA) software, SPSS (version 19.0) software (IBM Corporation, Armonk, NY), and R 3.5.1. *P *values were two‐sided, and *P* < 0.05 was defined as significant.

## 3 RESULTS

### 3.1 Baseline characteristics

Seventy‐six patients included in this study had a median age of 62 years, with a male predominance (male‐to‐female ratio, 3.0). Follow‐up data were available for 72 patients and the median follow‐up was 22.6 months (range: 0.9‐97.1 months). Sixty‐three patients had stage 4 disease (82.9%) and 56 patients (73.7%) had bone marrow involvement. Base on sMIPI, 29 (38.2%), 22 (28.9%), and 25 (32.9%) patients are classified as low‐risk, intermediate‐risk, and high‐risk patients, respectively. The median value of RDW was 14.4% (range: 12.0%‐21.9%) and 21 patients (21/76, 27.6%) had an abnormally elevated RDW (>15.7%) according to the normal reference in our hospital.

### 3.2 Association and correlation of RDW with other variables

**Figure 1:**
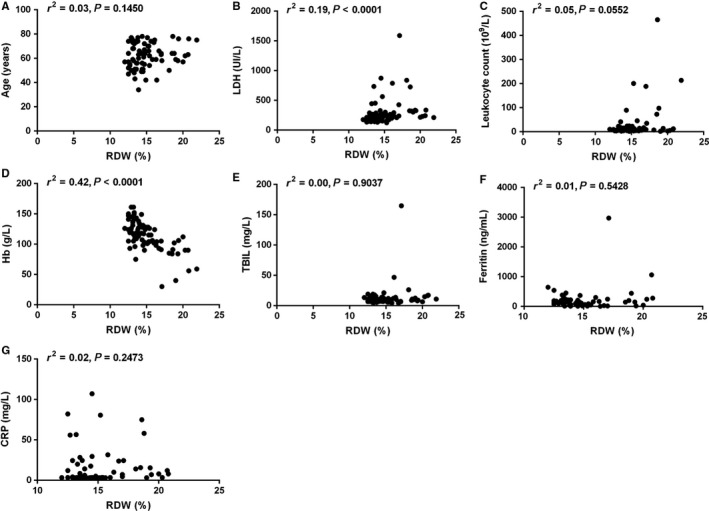
Correlation between RDW (red blood cell distribution width) and age (A), lactate dehydrogenase (LDH) (B), leukocyte (C), hemoglobin (Hb) (D), total bilirubin (TBIL) (E), ferritin (F), or C‐reactive protein (CRP) (G)

Association between RDW and other baseline variables was summarized in Table 1. RDW >15.7% was significantly associated with presence of B symptoms (*P* = 0.0020), elevated LDH (*P* = 0.0010), higher sMIPI (*P* = 0.0194), higher leukocyte count (*P* = 0.0345), and lower level of Hb (*P* < 0.0001). There was a trend that elevated RDW was associated with elevated CRP (*P* = 0.0862), although without significance. Further correlation analysis revealed that the value of RDW was inversely correlated with Hb level (*r* ^2^ = 0.42) and positively correlated with LDH level (*r* ^2^ = 0.19). The value of RDW was not correlated with age, leukocyte, TBIL, ferritin, or CRP (Figure 1).

**Table 1:** Baseline characteristics in patients with and without elevated RDW

Characteristic	Total, n = 76	RDW ≤15.7% (n = 55)	RDW >15.7% (n = 21)	*P* value^a^
Age^b^	62 (34‐78)	63 (42‐78)	60 (34‐78)	0.2556
Gender, no. (%)				
Male	57 (75.0)	43 (78.2)	14 (66.7)	0.3763
Female	19 (25.0)	12 (21.8)	7 (33.3)	
Stage, no. (%)				
1	1 (1.3)	1 (1.9)	0 (0.0)	0.2117
2	4 (5.3)	2 (3.7)	2 (9.5)	
3	7 (9.3)	7 (13.0)	0 (0.0)	
4	63 (84.0)	44 (81.5)	19 (90.5)	
B symptoms no. (%)				
Absent	36 (48.0)	32 (59.3)	4 (19.0)	0.0020
Present	39 (52.0)	22 (40.7)	17 (81.0)	
ECOG no. (%)				
<2	61 (80.3)	47 (85.5)	14 (66.7)	0.1042
≥2	15 (19.7)	8 (14.5)	7 (33.3)	
Number of extranodal sites				
≤1	47 (62.7)	35 (64.8)	12 (57.1)	0.5998
>1	28 (37.3)	19 (35.2)	9 (42.9)	
Elevated LDH no. (%)				
No	55 (72.4)	46 (83.6)	9 (42.9)	0.0010
Yes	21 (27.6)	9 (16.4)	12 (57.1)	
Leukocyte count (10^9^/L)	8.0 (2.0‐465.2)	7.3 (2.0‐200.7)	12.6 (2.0‐465.2)	0.0345
Simplified MIPI				
Low	29 (38.2)	25 (45.5)	4 (19.0)	0.0194
Intermediate	22 (28.9)	17 (30.9)	5 (23.8)	
High	25 (32.9)	13 (23.6)	12 (57.1)	
Hb (g/L)	115 (30‐161)	124 (75‐161)	91 (30‐124)	<0.0001
TBIL (mg/L)	9.8 (4.2‐164.7)	9.6 (5.1‐21.2)	10.9 (4.2‐164.7)	0.2915
Ferritin (ng/mL)	134 (5.3‐2971)	111.5 (8‐644)	169.9 (5.3‐2971)	0.2026
Elevated CRP				
No	40	32 (69.6)	8 (44.4)	0.0862
Yes	24	14 (30.4)	10 (55.6)	

Abbreviations: CRP, C‐reactive protein; ECOG PS, Eastern Cooperative Oncology Group performance status; Hb, hemoglobin; LDH, lactate dehydrogenase; MIPI, Mantle Cell International Prognostic Index; RDW, red blood cell distribution width; TBIL, total bilirubin.

a Comparison between patients with RDW ≤15.7% and RDW >15.7%.

b Median and range were reported for continuous variables.

### 3.3 Prognostic value of RDW and other factors in patients with MCL

**Figure 2:**
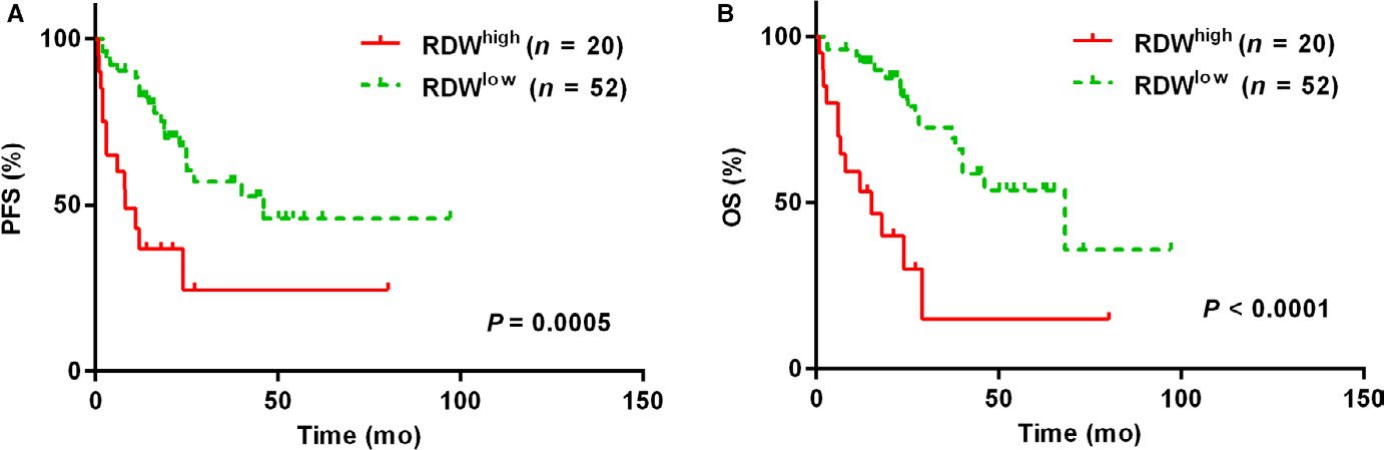
Progression‐free survival (PFS) (A) and overall survival (OS) (B) in patients with mantle cell lymphoma according to RDW (red blood cell distribution width). Abbreviations: RDW^high^, high RDW; RDW^low^, low RDW

The prognostic value of RDW was explored in our cohort. By using the X‐tile software, we found that 15.8% was the optimal cutoff that showed the most significant prognostic effects in predicting both OS and PFS. Therefore, we divided our patients with survival data into two groups： one group with high RDW (RDW >15.8%，n = 20） and one group with low RDW (RDW ≤15.8%，n = 52). We found that RDW >15.8% significantly predicted shorter PFS (hazards ratio [HR]: 3.14, 95% confidence interval [CI]: 2.08‐12.69; *P* = 0.0005) and shorter OS (HR: 4.04, 95% CI: 3.23‐22.82; *P* < 0.0001) (Figure 2). The impact of other factors on PFS and OS was also analyzed. We found that presence of B symptoms, elevated LDH, high sMIPI score, increased leukocyte count, and anemia were significantly associated with decreased PFS and OS (Table 2). Age >60 predicted shorter OS but not PFS. After multivariate analysis, we found that high RDW was the only factor that independently predicted both shorter PFS and OS (Table 3). And sMIPI was an independent prognostic factor with marginal statistical significance in predicting OS (Table 3).

**Table 2:** Association between Baseline Variables and Outcomes

	PFS			OS		
	HR	95% CI	*P*	HR	95% CI	*P*
High RDW	3.14	2.08‐12.69	0.0005	4.04	3.23‐22.82	<0.0001
ECOG PS ≥ 2	2.64	1.48‐10.44	0.0070	2.55	1.31‐10.05	0.0141
B symptom	2.50	1.28‐5.03	0.0091	2.95	1.57‐6.32	0.0016
Elevated LDH	3.90	2.89‐16.2	<0.0001	5.02	4.35‐27.4	<0.0001
High‐risk sMIPI	2.71	1.52‐6.95	0.0026	3.72	2.22‐11.37	0.0001
Leukocyte count ≥10×10^9^/L	2.54	1.41‐6.56	0.0050	3.01	1.66‐8.24	0.0015
Anemia	2.16	1.11‐4.36	0.0269	2.97	1.54‐6.63	0.0024
Age >60	1.67	0.84‐3.29	0.1498	2.28	1.11‐4.64	0.0272

Abbreviations: ECOG PS, Eastern Cooperative Oncology Group performance status; HR, hazards ratio; LDH, lactate dehydrogenase; OS, overall survival; PFS, progression free survival; RDW, red blood cell distribution width; sMIPI, simplified Mantle Cell International Prognostic Index.

**Table 3:** Multivariate Cox Analysis for PFS and OS

	PFS			OS		
	HR	95% CI	*P*	HR	95% CI	*P*
High RDW	2.26	1.00‐5.10	0.0493	2.99	1.28‐7.03	0.0118
B symptom	1.37	0.57‐3.33	0.4833	1.90	0.73‐5.00	0.1912
High‐risk sMIPI	1.92	0.88‐4.21	0.1030	2.22	0.97‐5.05	0.0578
Anemia	1.38	0.61‐3.13	0.4381	1.90	0.80‐4.55	0.1476

Abbreviations: HR, hazards ratio; OS, overall survival; PFS, progression free survival; RDW, red blood cell distribution width; sMIPI, simplified Mantle Cell International Prognostic Index.

### 3.4 RDW refines the prognostication by sMIPI

**Figure 3:**
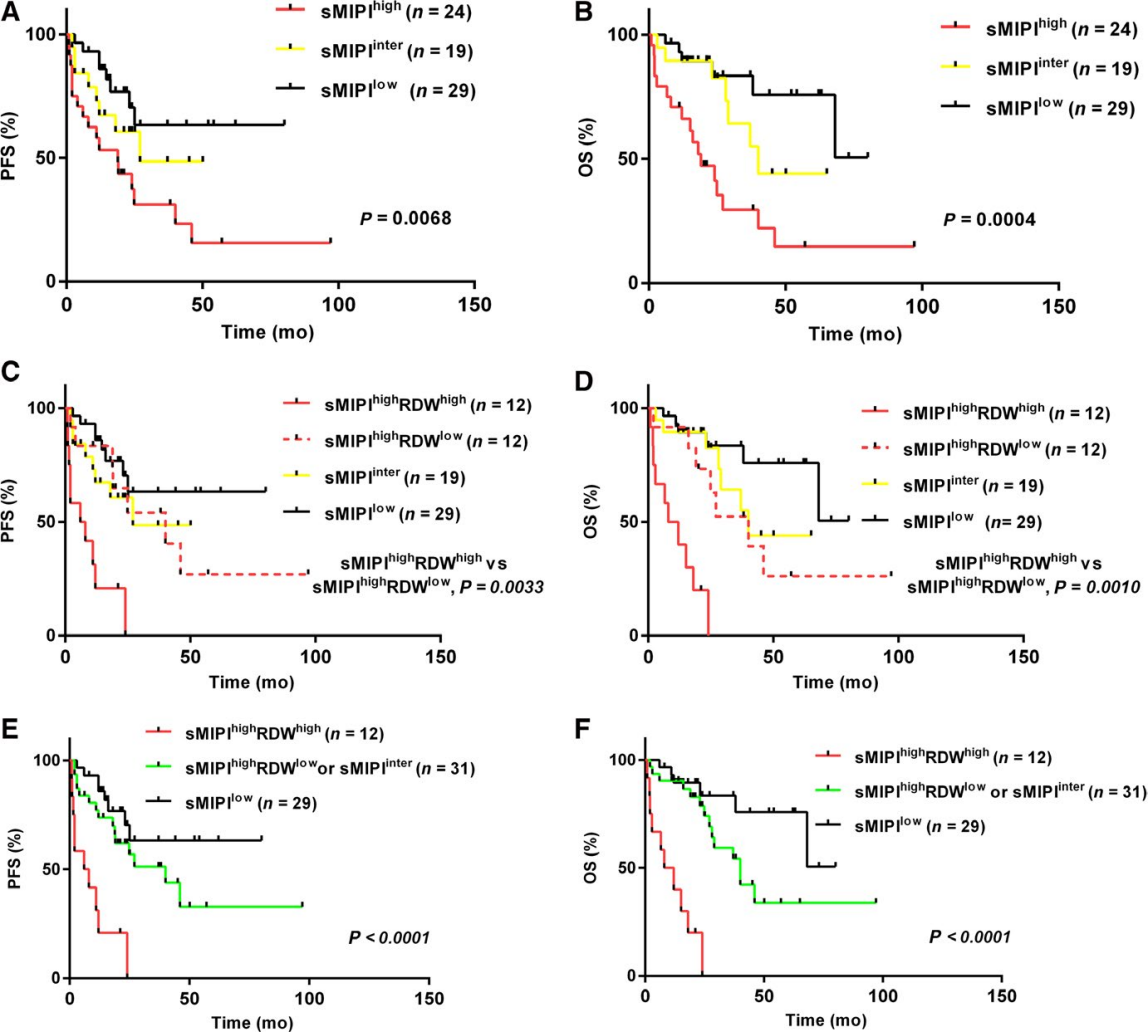
Progression‐free survival (PFS) (A) and overall survival (OS) (B) according to simplified Mantle Cell International Prognostic Index (sMIPI); progression‐free survival (C) and overall survival (D) stratified by red blood cell distribution width (RDW) in patients with high‐risk sMIPI; new stratification of progression‐free survival (E) and overall survival (F) according to sMIPI and RDW. Abbreviations: RDW^high^, high RDW; RDW^low^, low RDW; sMIPI^high^, high‐risk sMIPI; sMIPI^inter^, intermediate‐risk sMIPI; sMIPI^low^, low‐risk sMIPI

As a classical prognostic tool, sMIPI can stratify these patients into groups with different risk groups (Figure 3A,B). Patients in different groups based on sMIPI have significantly different PFS (*P* = 0.0068) and OS (*P* = 0.0004) (Figure 3A,B). We then explored if RDW could improve the prognostic value of sMIPI. We found that in high‐risk patients based on sMIPI, the patients with high RDW had a significantly shorter PFS (HR: 3.47, 95% CI: 1.95‐15.78; *P* = 0.0033) and OS (HR: 3.90, 95% CI: 2.48‐21.19; *P* = 0.0010) than those with low RDW (Figure 3C,D). High RDW did not have an impact on the outcome in patients with low‐risk sMIPI or intermediate‐risk sMIPI (data not shown). Further analysis revealed that patients with high‐risk sMIPI and low RDW had similar PFS (*P* = 0.97) and OS (*P* = 0.4889) to that of patients with intermediate‐risk sMIPI (Figure 3C,D). Therefore, we further categorize the patients in our cohort into three groups: patients with low‐risk sMIPI, patients with intermediate‐risk sMIPI or patients with high‐risk sMIPI and low RDW, and patients with high‐risk sMIPI and high RDW (Figure 3E,F). This new categorization can better classify these patients into groups with more significantly different PFS (*P* < 0.0001) and OS (*P* < 0.0001).

## 4 DISCUSSION

Mantle cell lymphoma is a heterogeneous disease in terms of morphology, clinical course, response to therapy and long‐term survival. An efficient prognostic tool is needed to facilitate clinical decisions. In addition to MIPI or sMIPI, biological factors have also been incorporated into the risk stratification of patients with MCL. Ki67 index, which is used to evaluate the proliferative capacity of tumor cells, has been demonstrated to be of prognostic value in MCL patients. Ki67 ≥30% is associated with decreased PFS and OS in patients with MCL,^8^ irrespective of the morphology subtypes. Combination of Ki67 with MIPI further refines the risk stratification based on MIPI, reflecting a stronger prognostic effect.^8^ Nevertheless, substantial variability in Ki67 scoring exists among different laboratories even the most experienced ones.^9^ Although Ki67 reproducibility can be improved by using digital image analysis, this technique is unavailable in most pathology laboratories.^10^ Other biological factors including SOX11 expression and TP53 mutation have been investigated. The prognostic role of SOX11 is not well‐established, with different studies showing different conclusions.^11^, ^12^ TP53 mutation has been identified a prognostic factor that predicts poor outcome in MCL patients.^13^, ^14^ However, the mutation status of TP53 is not routinely examined in most pathology laboratories.

In this study, we identified RDW, a clinically feasible marker, as a novel prognostic factor in MCL. Abnormally elevated RDW was associated with presence of B symptoms, elevated LDH level, increased leukocyte count, higher MIPI score, and decreased Hb level. Survival analysis revealed that high RDW (>15.8%) predicted both poor PFS and OS. More importantly, high RDW was demonstrated to be an independent predictor of both shorter PFS and OS. Further analysis suggested that it could stratify patients with high‐risk sMIPI score into two groups with different outcomes and improve the prognostic system based on sMIPI. Therefore, RDW can be used as a novel prognostic tool in MCL.

Both diagnostic and prognostic roles of RDW have been studied in cancer. Elevated RDW is more frequent in malignant biliary obstruction than benign cases, pointing to a diagnostic role of RDW in cases of biliary obstruction.^15^ Patients with urothelial carcinoma of the bladder (UCB) have elevated RDW compared with healthy individuals, suggesting RDW could be used in the diagnosis of UCB.^16^ Elevated RDW also helps identify colorectal cancer,^17^ ovary cancer,^18^ and myelodysplastic syndromes.^19^

The association between high RDW and poor prognosis has been found in a variety of solid tumors including esophageal cancer,^20^ rectal cancer,^21^ hilar cholangiocarcinoma, 22 and so on.^23^ The combinations of RDW with other factors have also been used to predict prognosis in patients with solid tumors.^24^, ^25^, ^26^ For hematological cancers, high RDW has been recognized as a poor prognostic factor in multiple myeloma, diffuse large B cell lymphoma, and chronic lymphocytic leukemia.^5^, ^27^, ^28^, ^29^, ^30^, ^31^, ^32^ The mechanism underlying the phenomenon that high RDW predicts poor outcome in cancer patients remains elusive. A possible explanation is that high RDW correlates with presence of severe systemic inflammation state and malnutrition, which are associated with poor outcome in cancer patients.^33^

Twenty‐one patients (27.6%) had an elevated RDW in our cohort. We tried to identify the causes of elevated RDW in our cohort. We found that elevated RDW was not associated with increased TBIL, suggesting elevated RDW was not caused by hemolytic anemia. We also found that elevated RDW did not have an association with low level of ferritin, indicating that iron deficiency was not a cause for RDW elevation in our cohort. There was a trend that higher CRP was associated with elevated RDW, although without statistical significance, suggesting elevated RDW might been caused by the presence of overwhelmed systemic inflammation.

In conclusion, our study demonstrated that high RDW predicted a poor outcome in patients with MCL. To our knowledge, it is the first study that suggests a prognostic role of RDW in MCL. It is an independent prognostic factor and also improves the prognostic stratification based on sMIPI. More efforts are needed to overcome the poor prognosis of MCL patients with high RDW.

## CONFLICT OF INTEREST

There is no conflict of interest.

## AUTHOR CONTRIBUTIONS

YM, JJG, WX, and JYL designed the study and wrote the manuscript. XHZ, QS, KS, JZW, HYZ, LW, and LF collected the data and did the analysis. All authors approved the final version.
